# Death of *Mycobacterium tuberculosis* by l-arginine starvation

**DOI:** 10.1073/pnas.1813587115

**Published:** 2018-09-06

**Authors:** Valerie Mizrahi, Digby F. Warner

**Affiliations:** ^a^South African Medical Research Council, National Health Laboratory Service, University of Cape Town Molecular Mycobacteriology Research Unit, University of Cape Town, 7925 Cape Town, South Africa;; ^b^Department of Science and Technology/National Research Foundation Centre of Excellence for Biomedical TB Research, Institute of Infectious Disease and Molecular Medicine, University of Cape Town, 7925 Cape Town, South Africa;; ^c^Department of Pathology, University of Cape Town, 7925 Cape Town, South Africa;; ^d^Wellcome Centre for Infectious Diseases Research in Africa, University of Cape Town, 7925 Cape Town, South Africa

Tuberculosis (TB) is currently the leading cause of mortality from a single infectious agent, resulting in more than 1.5 million deaths annually. In 2016, 10.4 million people developed the disease (1), of whom 490,000 had multidrug-resistant TB, defined as resistant to the two first-line drugs, isoniazid (INH) and rifampicin. Given that combination chemotherapies against the causative agent, *Mycobacterium tuberculosis* (*Mtb*), form the cornerstone of TB control, these stark statistics underscore the urgent need for new drugs to tackle this global health scourge. In response to this need, a pipeline has been established that has begun to deliver new and repurposed TB drugs (2). Key attributes for new drugs include efficacy against drug-resistant as well as drug-sensitive TB and an ability to effect a rapid, relapse-free (i.e., sterilizing) cure. These requirements have placed a high premium on the identification and validation of new targets, which differ from those of existing TB drugs, coupled with the development of potent compounds that will kill *Mtb* rapidly upon target engagement. This is a tall order for a notoriously underresourced field of research (3).

**Fig. 1.**
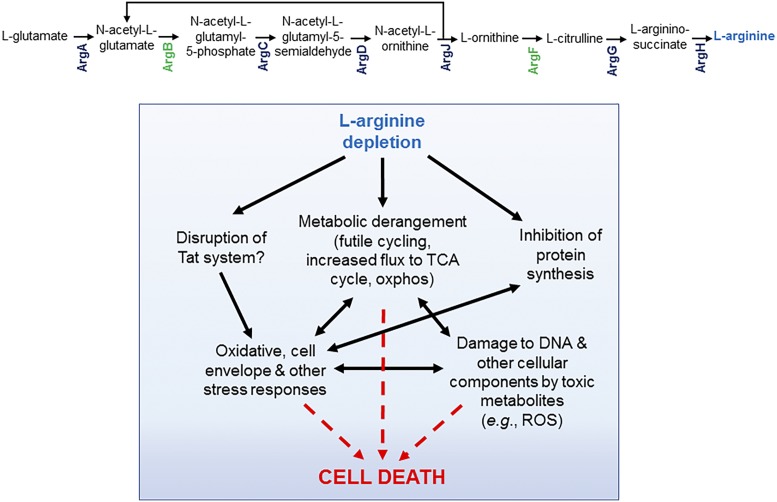
Death of *Mtb* resulting from l-arginine starvation is associated with complex metabolic disruption. The top section shows the biosynthetic pathway for l-arginine from l-glutamate, highlighting the steps at which pathway blockage occurs in the Δ*argB* and Δ*argF* mutants. Oxphos, oxidative phosphorylation; Tat, twin-arginine translocation; TCA, tricarboxylic acid.

In PNAS, Tiwari et al. (4) contribute to this endeavor by reporting the identification of two promising new TB drug targets: acetyl glutamate kinase (ArgB) and ornithine carbamoyl transferase (ArgF). These enzymes catalyze the second and sixth steps, respectively, in the pathway for the de novo biosynthesis of the amino acid l-arginine, from l-glutamate in *Mtb* (Fig. 1). The work of Tiwari et al. began with the observation that a feature common to the mycobactericidal agents, INH and vitamin C, is an ability to kill *Mtb* rapidly via a mechanism associated with the production of reactive oxygen species (ROS) (5). Using transcriptional profiling to investigate the effects of INH and vitamin C exposure on mycobacterial cellular metabolism, Tiwari et al. (4) discovered that up-regulation of several genes in the de novo pathway for l-arginine biosynthesis was a common early response of *Mtb* to both agents. This prompted the authors to investigate the impact of inactivating this biosynthetic pathway on bacillary function and survival. In addition to *argB*, Tiwari et al. chose *argF*, the subject of a prior study (6), for targeted gene knockout in *Mtb*. The resulting deletion mutants, Δ*argB* and Δ*argF*, were l-arginine auxotrophs, dependent on the exogenous supply of l-arginine at relatively high concentrations (>100 µM) for optimal growth in vitro. Notably, and in contrast to most other *Mtb* auxotrophs, the Δ*argB* and Δ*argF* mutants displayed a rapid loss of viability in the absence of supplement: in l-arginine–free media, cultures were completely sterilized within 20–40 d and showed no evidence of the emergence of escape mutants.

To explore the physiological basis of the rapid death of *Mtb* consequent on l-arginine starvation, Tiwari et al. (4) turned again to transcriptomics to monitor time-dependent changes in gene expression in the l-arginine–starved mutants over a period of up to 6 d. These studies give key insights into the nature and extent of the metabolic mayhem unleashed in *Mtb* upon l-arginine starvation over a period of time in which cell death—as evidenced by a decline in colony-forming units—was already underway. Up-regulation of genes in l-arginine biosynthesis, cell envelope stress and remodeling, oxidative stress and antioxidant defense, Fe-S cluster biogenesis and assembly, and DNA repair was observed in the Δ*argB* mutant under l-arginine starvation. l-Arginine biosynthesis, oxidative stress, and DNA repair genes were likewise induced in response to l-arginine starvation in the Δ*argF* mutant. Genes involved in biosynthesis of the antioxidant thiols, mycothiol and ergothioneine, were also up-regulated along with those involved in the production of the biosynthetic precursor, histidine. Moreover, while microaerobic respiration genes were up-regulated, this was accompanied by down-regulation of aerobic respiratory gene clusters and genes in regulons controlled by DosR (hypoxic and nitrosative stress), RelA (stringent response), and Rv1404 (acid stress). Based on these findings, Tiwari et al. (4) identify ROS-mediated oxidative damage as a likely culprit in the mechanism of l-arginine starvation-mediated cell death in *Mtb*.

To examine this association more closely, Tiwari et al. (4) used flow cytometry to measure the time-dependent accumulation of ROS and DNA damage in the Δ*argB* and Δ*argF* mutants in media with or without l-arginine supplementation. The 12- to 25-fold increase in endogenous ROS production in the l-arginine–starved mutants was found to correlate with increased levels of DNA damage. The mutants were also less able to withstand the toxic effects of hydrogen peroxide treatment under l-arginine starvation, showing enhanced ROS accumulation and hypersensitivity to this oxidizing agent. Providing further evidence in support of an association between l-arginine starvation, oxidative stress, ROS accumulation, and cell death, the rate of decline in mutant viability could be reduced by lowering the oxygen tension under which the cells were cultured.

To interrogate directly the consequences of arginine starvation in *Mtb* at the individual metabolite level, Tiwari et al. (4) performed metabolomic analyses on the Δ*argB* mutant over the same 6-d time course, sampling periodically to identify temporal changes in metabolite levels. Rapid and profound accumulation of the ArgB substrate, *N*-acetyl glutamate, was mirrored by a precipitous decline in the levels of the downstream metabolites, l-citrulline and l-arginine. The Δ*argF* mutant showed a similar response, with rapid accumulation of upstream metabolites accompanied by a decline in the levels of the ArgF product, l-citrulline, and the downstream metabolites, l-argininosuccinate and l-arginine, which dropped below their respective limits of detection after only 1 d. Other notable changes included depletion of ergothioneine and mycothiol, and accumulation of the secondary messenger, cAMP, in the Δ*argB* mutant. Together, these results are consistent with an emerging paradigm for bactericidal antibiotic lethality, whereby disruption of essential cellular processes resulting from the primary drug–target interaction is followed by metabolic derangement, stress responses, production of ROS, and other toxic metabolites, and ensuing damage of DNA and other cellular components, including nucleotides (7), ultimately leading to cell death (8) (Fig. 1). Less clear, however, is what sets l-arginine—and, to some extent, methionine (9)—apart from other essential metabolites (amino acids, vitamins, and cofactors) where deprivation is not as catastrophic for *Mtb*.

To assess the impact of l-arginine auxotrophy on *Mtb* pathogenesis, Δ*argB* and Δ*argF* mutants preloaded with l-arginine were deposited in the lungs of immunocompetent mice by the aerosol route and the course of infection monitored by scoring bacterial burdens in the infected animals over a period of 120 d. Both strains were highly attenuated in this model, with the organ bacillary loads dropping below the limit of detection after 3–4 wk, and lungs of the infected animals showing none of the histological signs of disease characteristic of mice infected with virulent *Mtb*. Further evidence of profound attenuation was provided by studies in severe combined immune-deficient mice using both low-dose

Tiwari et al. identify ROS-mediated oxidative damage as a likely culprit in the mechanism of l-arginine starvation-mediated cell death in *Mtb*.

aerosol and high-dose intravenous routes of infection; in both cases, the l-arginine auxotrophs failed to grow and cause fatal disease in these animals. That is, in all mouse models tested, the auxotrophs appeared unable to scavenge host-derived metabolites that could ameliorate the lethal effects of l-arginine starvation. These include l-arginine itself, as well as biosynthetic intermediates downstream of the step blocked by the absence of either ArgB or ArgF (Fig. 1). This conclusion is consistent with the notion of *Mtb* as a self-sufficient pathogen that does not depend on its host to provide the nutrients required to grow, survive, and cause disease (9). However, the discrepant in vivo phenotypes of the Δ*argF* mutant reported in this study, and that reported previously (6)—likely a double *argF–argG* deletion because of the insertional mutagenesis strategy employed—might offer some insight into the mechanisms regulating and enabling assimilation of l-arginine and its precursors.

ArgB and ArgF have thus joined the ranks of a growing number of new TB drug targets (10, 11), including ArgJ (12), another enzyme in the l-arginine pathway, as well as enzymes involved in the biosynthesis of other amino acids (9, 13, 14), vitamins (15), and cofactors (16). The rapid-death phenotype is one of several attributes to consider when ranking ArgB and ArgF against other targets that have been validated genetically using gene knockouts, as used here (4), or conditional knockdowns (17); in this context, it would be very interesting to determine the effect of depleting *argB* or *argF* during chronic infection, a model that more closely approximates the clinical situation than the use here of preloaded auxotrophs. Target vulnerability—the extent of target inactivation required to suppress growth—is another important attribute that has yet to be established for ArgB and ArgF, and could be determined using the same knockdown approach. For those genetically validated targets that are druggable, fragment-based drug design offers the prospect of developing potent chemical inhibitors. However, translating such molecules into antitubercular agents that can cross the cell envelope of *Mtb*, avoid expulsion by efflux, evade the formidable biotransformation machinery that *Mtb* can use to detoxify xenobiotics (18), and engage their targets, is a challenging proposition (19). A further consideration is that the bar for validating TB drug targets whose essentiality might be subverted by metabolite rescue has been raised by a recent study involving inosine 5′-monophosphate dehydrogenase (IMPDH) (20), a putative target in de novo purine biosynthesis. There, the importance of establishing metabolite levels in human tissue was underscored by the observation that guanine, a metabolite that can rescue *Mtb* from IMPDH inhibition via the purine salvage pathway, was present in much greater concentrations in normal lung tissue and caseum in rabbits and humans than in mice. These challenges notwithstanding, it is essential to proceed at pace in pursuing promising new lines of investigation, such as that established by Tiwari et al. (4). The magnitude of the problem confronting us demands nothing less.
